# Supplementary material for: Photocatalytic Degradation, Anticancer, and Antibacterial Studies of Lysinibacillus sphaericus Biosynthesized Hybrid Metal/Semiconductor Nanocomposites
Source: Microorganisms. 2023 Jul 14;11(7):1810. doi: 10.3390/microorganisms11071810 (PMC10385839; doi:10.3390/microorganisms11071810)
Supplement: Supplementary file 1 [file microorganisms-11-01810-s001.zip › microorganisms-2493887-supplementary.pdf]

Supplementary Materials

Photocatalytic Degradation, Anticancer, and Antibacterial Studies of *Lysinibacillus sphaericus* Biosynthesized Hybrid Metal/Semiconductor Nanocomposites

Kannan Badri Narayanan [1,2,*], Rakesh Bhaskar [1], Yong Joo Seok [1] and Sung Soo Han [1,2,*]

[1] School of Chemical Engineering, Yeungnam University, 280 Daehak-Ro, Gyeongsan 38541, Gyeongbuk, Republic of Korea; indiaxenobiotics@gmail.com (R.B.); badri.explore@gmail.com (Y.J.S.)
[2] Research Institute of Cell Culture, Yeungnam University, 280 Daehak-Ro, Gyeongsan 38541, Gyeongbuk, Republic of Korea
* Correspondence: okbadri@gmail.com or okbadri@yu.ac.kr (K.B.N.); sshan@yu.ac.kr (S.S.H.)

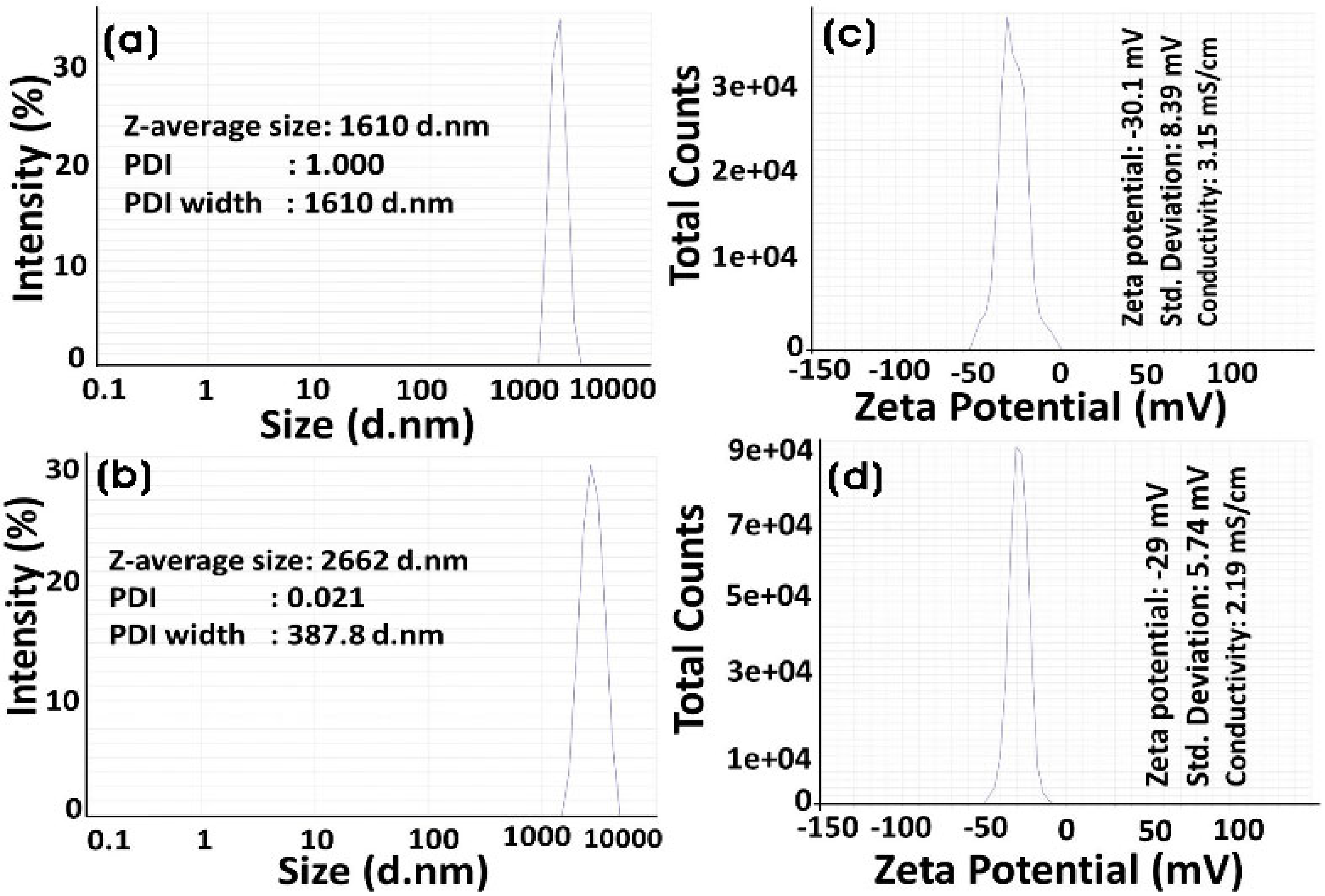

Supplementary Figure S1. (a, b) Dynamic light scattering (DLS) and (c, d) zeta (ζ) potentials of biosynthesized ZnO and Ag/ZnO NCs.

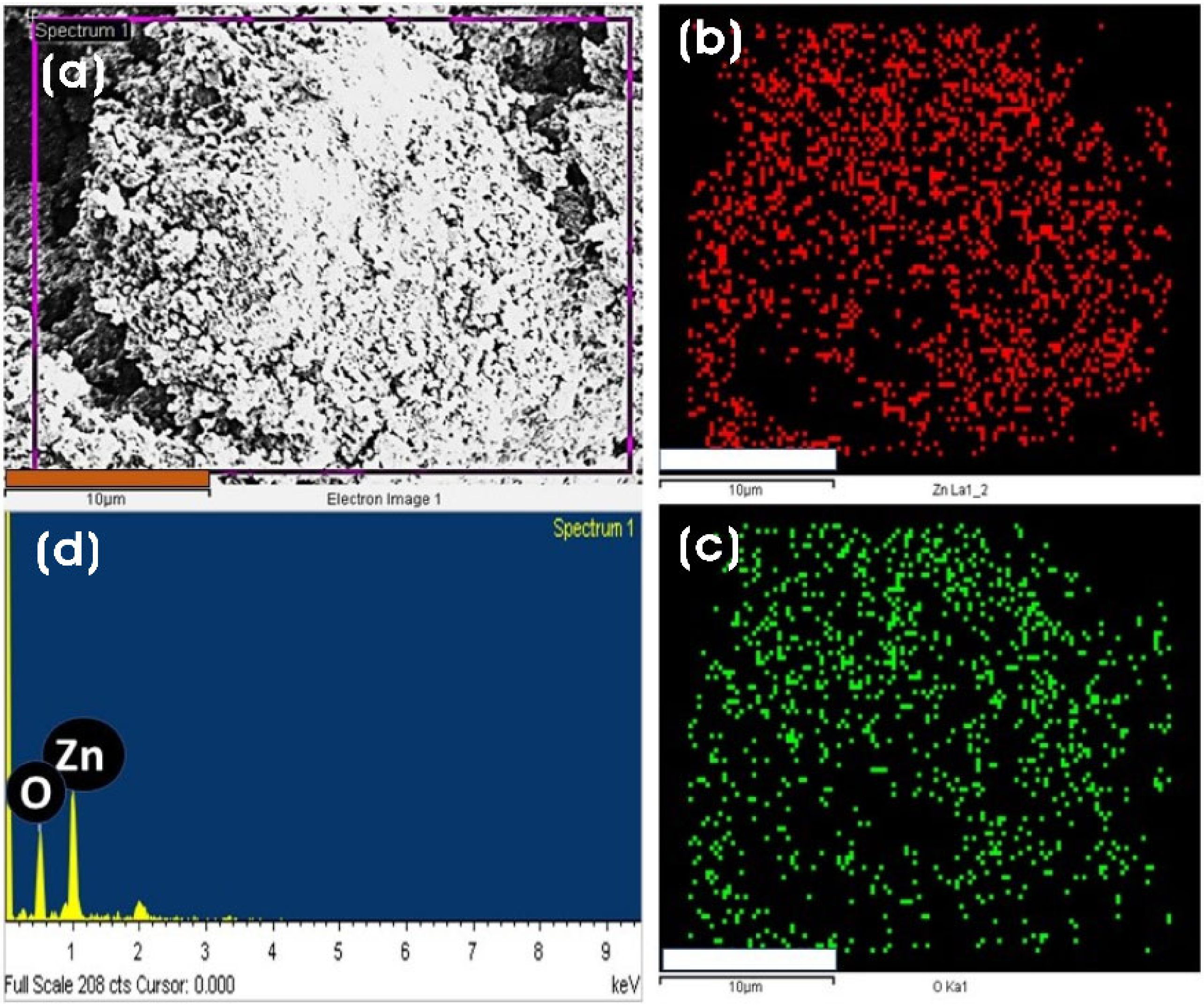

Supplementary Figure S2. FE-SEM-EDX analysis of ZnO. (a) FE-SEM micrograph and elemental maps of (b) Zn-L (c) O-K and (d) EDX spectrum.

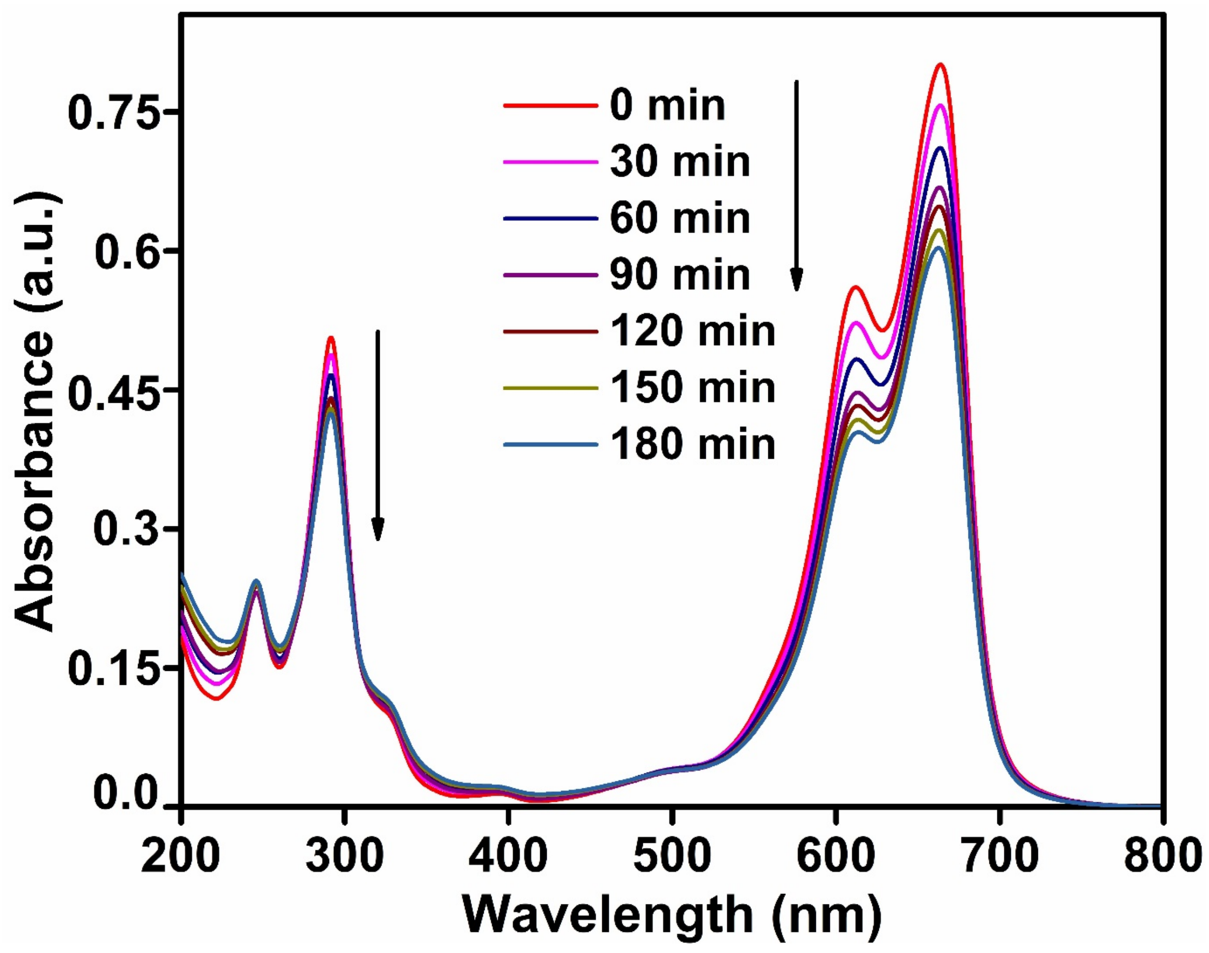

Supplementary Figure S3. Photolysis of methylene blue dye without any photocatalysts.

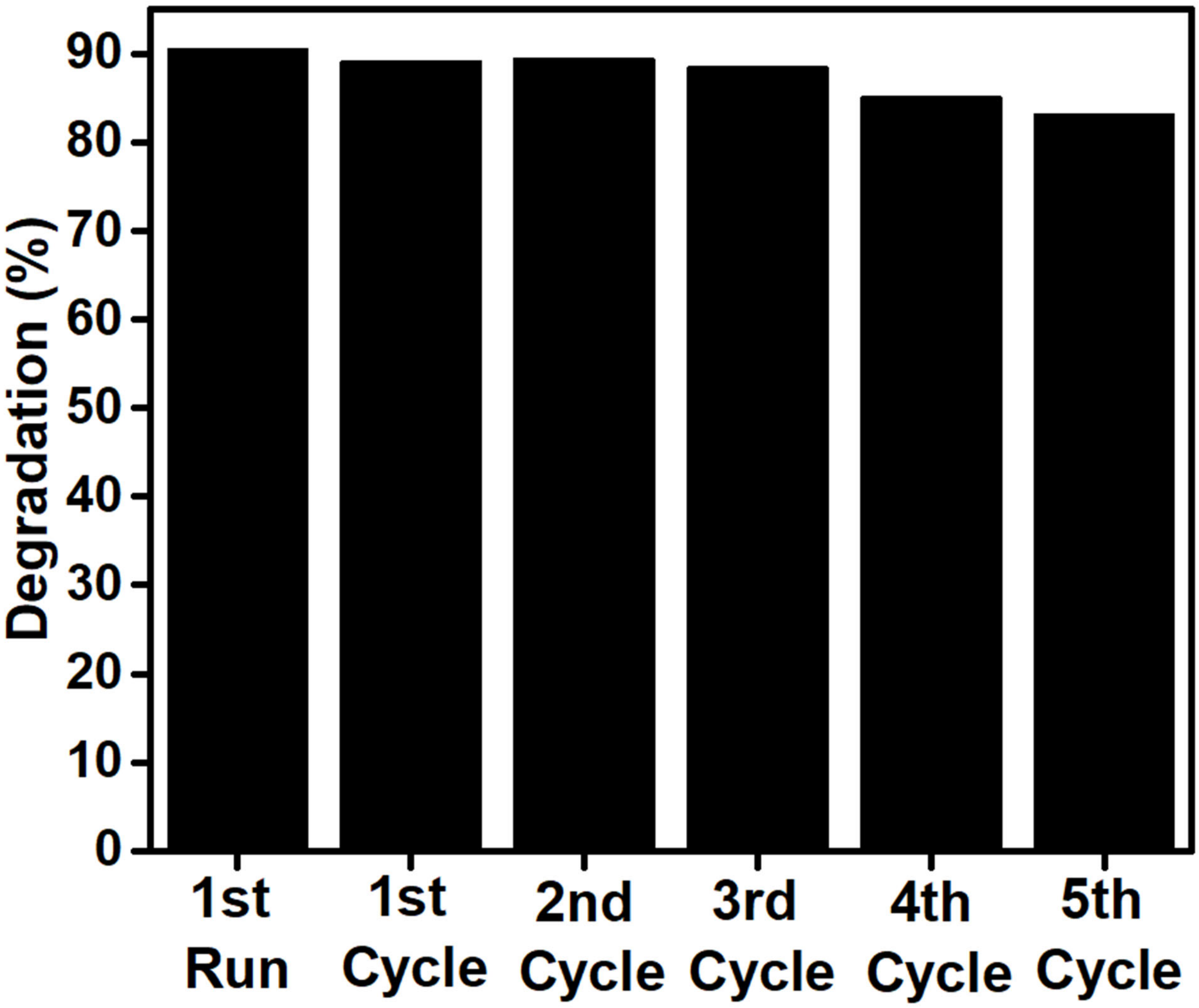

Supplementary Figure S4. Reusability of Ag/ZnO NC as a photocatalyst in the degradation of MB up to five cycles.
